# Cutaneous Adverse Reactions Associated with SARS-CoV-2 Vaccines

**DOI:** 10.3390/jcm10225344

**Published:** 2021-11-16

**Authors:** Francesco Bellinato, Martina Maurelli, Paolo Gisondi, Giampiero Girolomoni

**Affiliations:** Section of Dermatology and Venereology, Department of Medicine, University of Verona, Piazzale A. Stefani 1, 37126 Verona, Italy; maurelli.martina@gmail.com (M.M.); paolo.gisondi@univr.it (P.G.); giampiero.girolomoni@univr.it (G.G.)

**Keywords:** vaccines, COVID-19, cutaneous adverse reaction, exanthema, safety

## Abstract

Many patients are receiving SARS-CoV-2 vaccinations, which have been associated with a variety of adverse effects. Cutaneous adverse reactions to SARS-CoV-2 vaccinations have been progressively reported, but they have not been reviewed according to their morphological clinical patterns. The objective of this review was to summarize the existing data concerning the cutaneous adverse reactions following SARS-CoV-2 vaccines and group them according to common morphological and pathogenetic patterns. We reviewed the English language literature up to 15 August 2021, using predefined keywords to identify the relevant studies evaluating cutaneous adverse reactions associated with SARS-CoV-2 vaccines. We search for recurrent morphological patterns sharing clinical signs and symptoms and physio-pathological mechanisms. Timing to onset following the first or booster dose of the vaccine, predisposing conditions, therapeutic management, and outcome were also collected. Among the dermatological manifestations associated with SARS-CoV-2 vaccinations, we distinguished: (1) new onset reactions and (2) flares of preexisting dermatoses. The most common were injection site reactions, affecting 30–70% and generally mild or moderate. Small case series or single case reports included filler reactions, exanthemas, vascular lesions, urticaria, eczematous dermatitis, autoimmune bullous reactions, and severe cutaneous adverse reactions. In addition, the exacerbation of chronic immuno-mediated dermatoses (mainly psoriasis and atopic dermatitis) and reactivations of herpes infection were reported. The cutaneous reactions were generally mild, self-limiting, and resembled common cutaneous drug eruptions and/or COVID-19 skin manifestations.

## 1. Introduction

To stem the severe acute respiratory syndrome coronavirus 2 (SARS-CoV-2) pandemic, a very large vaccination campaign is spreading widely. A massive number of individuals are going to receive vaccinations globally. Different strategies are being explored as possible vaccines against SARS-CoV-2, including inactivated virus vaccines, virus-like particle or nanoparticle vaccines, protein subunit vaccines, virus-vectored vaccines, DNA and messenger RNA (mRNA) vaccines, and live-attenuated virus vaccines [1]. Single-stranded RNA (ssRNA) and double-stranded RNA (dsRNA) act as potent inflammatory signals. Upon inoculation, they are recognized by various endosomal and cytosolic innate sensors (i.e., TLR3, TLR7, and components of the inflammasome), resulting in cellular activation, the production of type I interferon, and multiple inflammatory mediators [2]. The potency of mRNA vaccines has been optimized by the encapsulation of the mRNA into lipidic nanoparticles, called LNPs, throughout the protection of the mRNA from degradation by RNase enzymes. The intramuscular administration of LNP-formulated mRNA vaccines results in local and transient inflammation in the muscle, which drives the recruitment of neutrophils and antigen-presenting cells (APCs) to the site of delivery [3]. Two mRNA (Pfizer/BioNTech BNT162b2 and Moderna mRNA-1273) and two adenovirus-vectored (AstraZeneca/Oxford AZD1222 and Johnson & Johnson/Janssen Ad26.CoV2.S) vaccines have already received conditional marketing authorization by the European Medical Agency (EMA). Control for vaccine safety requires a proactive approach, involving vigilance, response, documentation, and characterization of the events [4]. Cutaneous manifestations following vaccinations are not uncommon. As a consequence, a significant number of adverse skin reactions are expected to develop [5]. When several individuals will be immunized in mass vaccinations campaigns, even rare adverse events might be encountered more often. Clinicians should be prepared to address dermatological reactions (local and systemic) to SARS-CoV-2 vaccinations. The objective of this review was to summarize the existing data concerning the cutaneous adverse reactions following SARS-CoV-2 vaccines and group them according to common morphological and pathogenetic patterns.

## 2. Materials and Methods

We carried out a narrative review of the English language literature related to any cutaneous adverse reactions attributed to SARS-CoV-2 vaccines. The terms used for the PubMed search were as follows: "COVID-19 Vaccine*" OR "SARS-CoV-2 Vaccination*" AND "Skin*" OR "Cutaneous*" OR "delayed*" OR "dermatology*" OR "vesiculo*" AND "reaction*" OR "effect*" OR "event*" OR "Eruption*" OR "rash*" OR "flares*" OR "eczema*" OR "lesion*" OR "pemphigus*" OR "psoriasis*", NOT "thrombocytopenia*" OR "cardiological*" OR "neurological*" OR "myocarditis*" OR "thromboembolic*". The search included original articles (i.e., case reports, case series, registry-based observational studies, and randomized controlled trials) published from 31 March 2020 (inception date) to 15 August 2021.

There were no restrictions in terms of sex, race, or geographic area. Additionally, we reviewed references from relevant original papers and review articles to identify further eligible studies not covered by the original database search. Studies describing clinical cutaneous adverse reactions after at least one dose of mRNA (Pfizer/BioNTech BNT162b2 and Moderna mRNA-1273) or adenovirus-vectored (AstraZeneca/Oxford AZD1222 and Johnson & Johnson/Janssen Ad26.CoV2.S) SARS-CoV-2 vaccine were included. In the manuscripts, the association between cutaneous adverse reactions and SARS-CoV-2 vaccinations was supported by temporal and topographical criteria, the lack of any other trigger factors (i.e., infections or new medications), recurrence with the booster, previous published reports, and/or biological plausibility. The criteria for exclusion were as follows: (1) studies that did not have a clear morphologic description of the dermatologic signs and/or symptoms in their clinical presentations; (2) studies involving pediatric populations; (3) articles written in a language other than English; and (4) review articles, conference abstracts, and expert opinions. For all eligible studies, the following data were retrieved: authors and year of publication; study country; study design; sample size; population characteristics (age, sex, preexisting dermatologic, and/or allergic conditions); type of cutaneous reactions; timing to onset following the first or booster dose of the vaccine; histopathological assessment; therapeutic management; and outcome. To illustrate the clinical appearance of the patterns, the pictures of the original cases observed in our clinical practice were included.

## 3. Results

**Figure 1:**
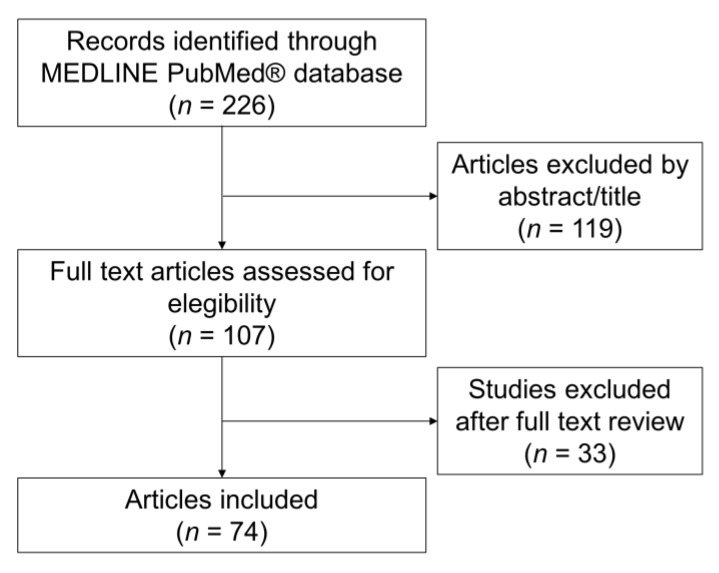
Screening flow chart.

A total of 226 articles were identified through PubMed Medline^®^. After screening through the titles and abstracts, 151 articles were excluded because they were not pertinent. A total of 107 articles underwent full-text assessments for eligibility, and 33 articles were excluded based on the exclusion/inclusion criteria. Finally, a total of 74 studies were included in the review (Figure 1). 

We classified the cutaneous adverse reactions associated with SARS-CoV-2 vaccinations into two groups: (1) new onset skin reactions and (2) flares of preexisting dermatoses. Among the former, we classified cutaneous reactions in different patterns sharing morphological and pathogenetic mechanisms as follows: injection site reactions (immediate and delayed local reactions/COVID arm); filler reactions; exanthemas (confluent erythematous rash, morbilliform or maculopapular, varicelliform or papulovesicular, pityriasis rosea/pityriasis rosea-like, symmetrical drug-related intertriginous and flexural exanthema (SDRIFE), and annular); urticaria (wheals and/or angioedema); vascular lesions (vasculitis, purpura/petechiae, livedo, acral chilblain-like, and erythromelalgia); eczematous dermatitis; autoimmune bullous diseases; and severe cutaneous adverse reactions (SCARs), including acute generalized exanthematous pustulosis (AGEP) and Stevens-Johnson syndrome/Toxic Epidermal Necrolysis (SIS/TEN). Each pattern is described below (Table 1).

### 3.1. Characteristics of Subjects Developing Cutaneous Adverse Reactions

According to the data retrieved from a large registry-based study and a national cross-sectional study, involving 819 reactions in 805 patients overall, adverse cutaneous reactions associated with SARS-CoV-2 vaccinations are more frequent among females than males, ranging from 80% to 90% of the reported cases [6,7]. Generally, cutaneous reactions are slightly more common after the first dose compared to the booster (53% vs. 46%, respectively) [6]. Recurrence after booster inoculation was reported in a proportion ranging from 8% to 46% of the patients who experienced a reaction after the first dose. In particular, Català et al. found that 8.5% (14) of patients with first dose reactions developed a second dose reaction, and in seven of them, it was the same [6]. The mean time to onset after the vaccination ranges from immediate reactions, occurring within hours after inoculation, to some weeks, as it might depend on several variables (type and dose of vaccine, immunological state, history of COVID-19, comorbidities, etc.). In these two large studies, the proportion of cases with a history of atopic dermatitis and chronic urticaria was low compared to the general population (4.1–6.9% vs. 10% and 1.7–6.4% vs. 20%, respectively), suggesting that such conditions may not predispose toward cutaneous adverse reactions, as is well-established for cutaneous drug reactions in general [6,7].

### 3.2. Injection Site Reactions

**Figure 2:**
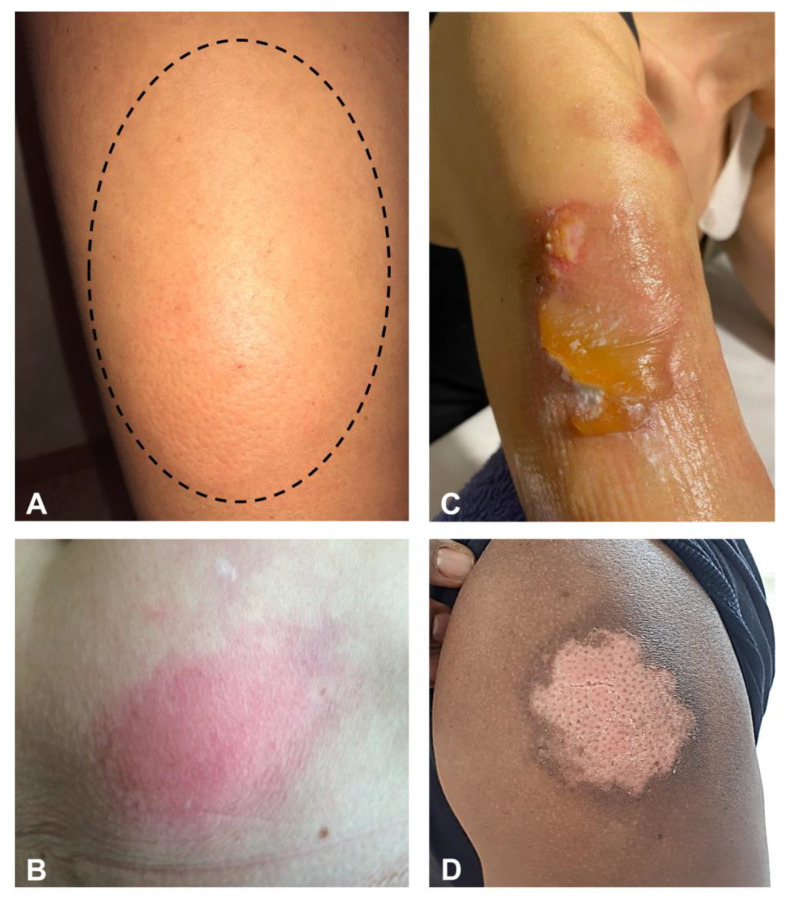
Edematous (**A**) and erythematous (**B**) indurated and tender plaques in the site of the injection developed one week after BNT162b2 vaccination in 36- and 67-year-old females, respectively. Burning bullous reactions on the deltoid area after the application of an ice pack to relieve the pain in a 56-year-old woman (**C**). Depigmented erythematous plaque of the deltoid skin developed in a 39-year-old African man three weeks after BNT162b2 vaccination (**D**).

Both immediate and delayed local reactions are extremely common. For example, in a national cross-sectional study involving a total of 415 cutaneous reactions, Català et al. found that 32.1% (130) were injection site reactions, of which 53.8% were delayed [6]. Immediate and delayed local reactions represented 52.4% (232) and 49.2% (218) of a large registry-based study of 443 reactions, respectively [7]. Tenderness, itchiness, erythema, and swelling may develop immediately up to a few days after inoculation (Figure 2A,B) [8,9]. Delayed reactions, occurring some days after vaccination, are even more common, representing more than half of the local reactions [6]. Similar findings were confirmed in Black, Indigenous, or People of Color [10]. The so-called COVID arm is an erythematous, indurated, edematous, itchy, or painful plaque of different sizes developing in the area where the vaccine was inoculated [11,12]. Necrotic lesions characterized by angulated eschars surrounded by indurated erythematous borders at site of inoculation were reported one week after BNT162b2 vaccination [13]. The mean time to onset ranges from 4 to 11 days from the first [7,8,14] and 2–5 days from the second dose [7]. The COVID arm can recur with the booster but is, on average, likely to be less severe and may develop faster [7]. In the case of fever, the suspect of cellulitis should be ruled out [12]. Histopathology shows a superficial and deep perivascular lymphocytic infiltrate with dilated vessels, intraluminal neutrophils, and negative immunohistochemistry for the SARS-CoV-2 spike protein, consistent with delayed-type or T-cell-mediated hypersensitivity reactions [12]. Symptomatic therapies as antihistamines and/or topical glucocorticoids can be used for treatment, as for drug-induced injection site reactions [14,15]. No serious adverse reactions have been reported after the second dose. Burning bullous reactions have been observed after the application of an ice pack for a relief from pain (Figure 2C). Pigmentary changes (hyper or hypo) may follow the acute phase, especially after more severe reactions (Figure 2D).

### 3.3. Filler Reactions

Interestingly, unusual delayed inflammatory reactions to dermal hyaluronic acid filler employed for aesthetics were described both following COVID-19 infection and mRNA vaccinations. The clinical presentation is characterized by slightly erythematous, soft, and tender swelling of the filler injection site [7,16,17]. Filler reactions can be auto-resolutive or symptomatic, requiring hyaluronidase injections and systemic glucocorticosteroids. Since a potential pathophysiologic mechanism of filler reactions is the binding of spike protein with dermal ACE2 receptors, some authors have treated this reaction with low-dose angiotensin-converting enzyme inhibitors to reduce proinflammatory angiotensin II [14].

### 3.4. Exanthemas

**Figure 3:**
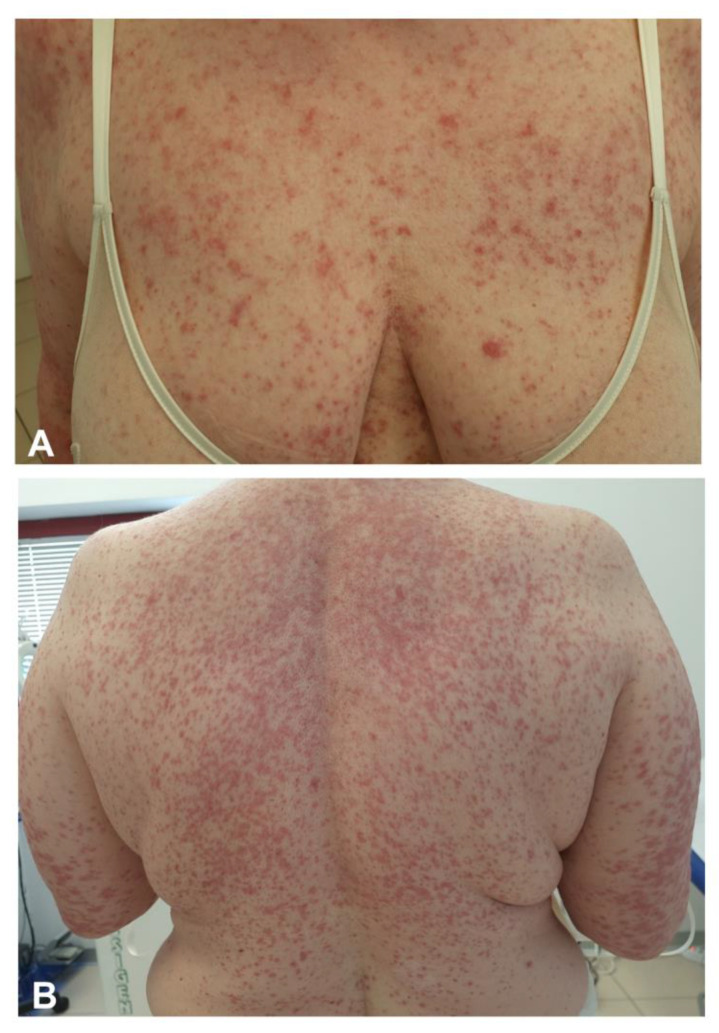
Itchy confluent erythematous maculopapular eruption involving the upper trunk and limbs in a bilateral and symmetrical fashion and typical craniocaudal progression developed five days after the mRNA-1273 vaccine on a 54-year-old woman (**A**,**B**).

**Figure 4:**
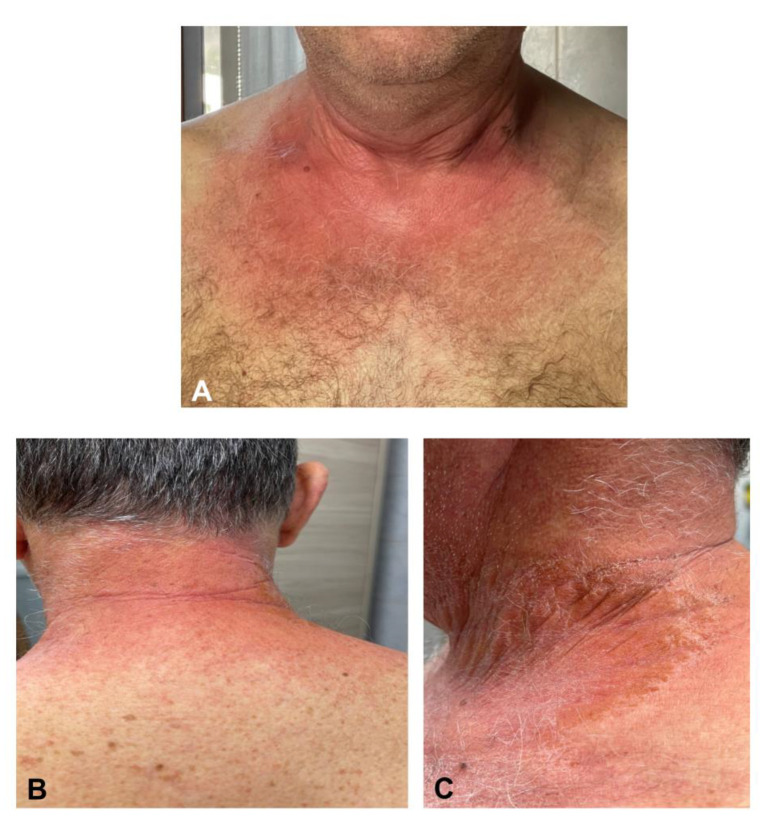
Erythematous and sharply demarcated lesions with symmetric and intertriginous-flexural distribution involving the neck and the gluteal area (symmetrical drug-related intertriginous and flexural exanthema—SDRIFE) developed on a 65-year-old physician two weeks after the BNT162b2 vaccine (**A**–**C**).

Exanthemas following SARS-CoV-2 vaccination can develop either as a pruritic confluent erythematous or maculopapular morbilliform eruption generally involving the face, the trunk, and the limbs in a bilateral and symmetrical fashion with a typical craniocaudal progression (Figure 3). Histopathology has revealed classical findings, i.e., mixed-cell infiltrate with eosinophils, epidermal spongiosis, keratinocyte apoptosis, and vacuolar interface changes [6]. Pruritus may be severe. A purpuric hue can be found, especially when the rash involves the inferior limbs [18,19,20,21,22,23]. A -papulovesicular or varicelliform rash is characterized by small papules/vesicles surrounded by erythema without a herpetiform arrangement [6]. In a large cross-sectional study involving a total of 415 cutaneous reactions, the prevalence of morbilliform and papulovesicular rashes was 8.9% (36) and 6.4% (26), respectively [6]. McMohan et al. observed similar proportions, reporting a prevalence of 6.1% (27 out of 443) morbilliform and 8.3% (10 out of 443) papulovesicular rashes, respectively [7]. The maculopapular rash develops in almost 70% of cases after the first dose, with a timing to onset ranging from two days to two weeks [6]. In a large prospective cohort study of almost 50,000 health employees who received mRNA vaccines, itchy exanthema was the most common cutaneous reaction, reported by 1.5% of the patients after the first dose and 1.6% after the second [8]. In a randomized, cross-sectional study of 803 patients after BNT162b2 vaccination, the self-reported prevalence of exanthema was 2.5% [24]. Recurrence occurred in 16% of those who experienced the first reaction. Exanthema reactions can be easily treated with systemic and/or topical corticosteroid and systemic antihistamines to relieve symptoms and shorten the disease duration [20,21]. We also observed the occurrence of uncommon rashes as SDRIFE (Figure 4). Rarely, an extensive erythematous rash with blistering evolution may occur [25]. Atypical rashes reminiscent of pityriasis rosea following 4–7 days after mRNA vaccination have also been reported [6,7,19,20,25,26]. Unlike pityriasis rosea, these reactions are characterized by more itchy and diffuse lesions with a more frequent involvement of mucous membranes and no preceding herald patch [27]. It is still not clear whether pityriasis rosea-like exanthema is secondary to the direct invasion of SARS-CoV-2 or HHV-6/7 reactivation [27]. One case of figurate annular eruption following Ad26.CoV2.S vaccine was also reported [28].

### 3.5. Vascular Lesions

Distinct types of vascular skin lesions have been described, including leucocytoclastic vasculitis, purpura/petechiae, livedo, chilblain-like lesions, and erythromelalgia.

Cases of cutaneous leukocytoclastic vasculitis were described following both the first and booster doses of mRNA and adenovirus-vectored vaccinations, including vasculitis urticaria [6,7,9,29,30]. In a national cross-sectional study involving a total of 415 cutaneous reactions, Català et al. found that 4% (*n* = 16) were a purpuric rash of the lower limbs, of which four were cutaneous leukocytoclastic vasculitis [6]. The clinical presentation showed the typical features of cutaneous small-vessel vasculitis, including confluent palpable purpura lesions in the buttocks, legs, lower portion of the abdomen, and arms. The histopathological findings included perivascular and interstitial neutrophilic infiltrate with leukocytoclasia and fibrin deposition within vessel walls [20,30]. The time to onset ranged from 2 to 7 days after vaccine inoculation. The new onset of urticarial vasculitis was also described 5 days following a second dose of the BNT162b2 vaccine [23]. Small-vessel vasculitis has been described after several vaccination types—in particular, the influenza vaccine [31].

Improvement of the lesions can be obtained after a course of systemic and topical glucocorticosteroids. Petechial eruptions were described as a possible consequence of immune thrombocytopenic purpura following mRNA vaccination. Some cases were accompanied by systemic signs/symptoms like weakness, shortness of breath, leg edema, nausea, vomiting, and abdominal pain [32,33,34]. A petechial rash of the trunk was even reported in a patient with anaphylactic symptoms [35]. Other forms of purpuric/petechial lesions can be the expression of eczematous pigmented purpura [6] or presenting as an asymptomatic purpura of the eyelid [36] or a brownish rust-like macular erythema of the hands, possibly due to hemosiderin deposition [37]. Purpura can also be arranged in “whip-like" linear or curvilinear streaks as purpura flagellate [38]. Livedo racemosa of the thighs is another vascular presentation following the mRNA vaccine [18].

Sars-CoV-2 vaccine-related chilblain-like lesions are painful and have itching violaceous nodules in acral sites (toes and hands) resembling pernio and COVID toe. Chilblain-like lesions can develop in 4–12 days after the first dose of mRNA or inactivated SARS-CoV-2 vaccines (CoronaVac by Sinovac Life Sciences) [6,19,23,39,40,41]. Topical glucocorticosteroids and antihistamines have been used with success [23].

Interestingly, erythromelalgia was reported in 14 patients out of 414 (median age was 38 years (range 19–83)) of one large registry-based study [7]. Lesions usually develop within an average of 7 days after the first dose and earlier after the booster. The common reported sites of involvement are the arms (69%), face (31%), hands (23%), and feet (15%). Farinazzo et al., Corbeddu et al., and Al-Ansari R et al. described cases of itchy acral erythema and swelling limited to the palms, hands, feet, and/or arms following the BNT162b2 vaccination in middle-aged women [19,42,43]. 

### 3.6. Urticaria/Angioedema

Acute urticaria presenting with itchy wheals and/or angioedema can develop immediately, i.e., within hours after vaccine injection, or later, after some days [23]. Among 5574 patients receiving the first dose of BNT162b2, Bianchi et al. found that 0.1% developed urticaria or angioedema within 4 h [44]. Conversely, an eruption that developed after 24 h in 92% of the cases of urticaria reported by McCahon et al. (37) and in 93% of the cases reported by Català et al. (29) [6,7]. Robinson et al. reported a prevalence of urticaria and angioedema following the first dose of the mRNA vaccination of 0.3% and 0.2%, respectively, and 0.6% and 0.4% following the booster [8]. The recurrence of urticaria and angioedema occurred in 3.3% and 2.6% of those who experienced the first reaction, respectively. Among 803 patients receiving the BNT162b2 vaccination, Kadali et al. described wheals and angioedema of the mouth/throat in 0.6% and 0.12–0.37% of the cases, respectively [24]. Treatment with H1 antihistamines is generally employed successfully to resolve the lesions, and systemic glucocorticoids can be used in the case of persistence [45].

### 3.7. Eczematous Dermatitis

**Figure 5:**
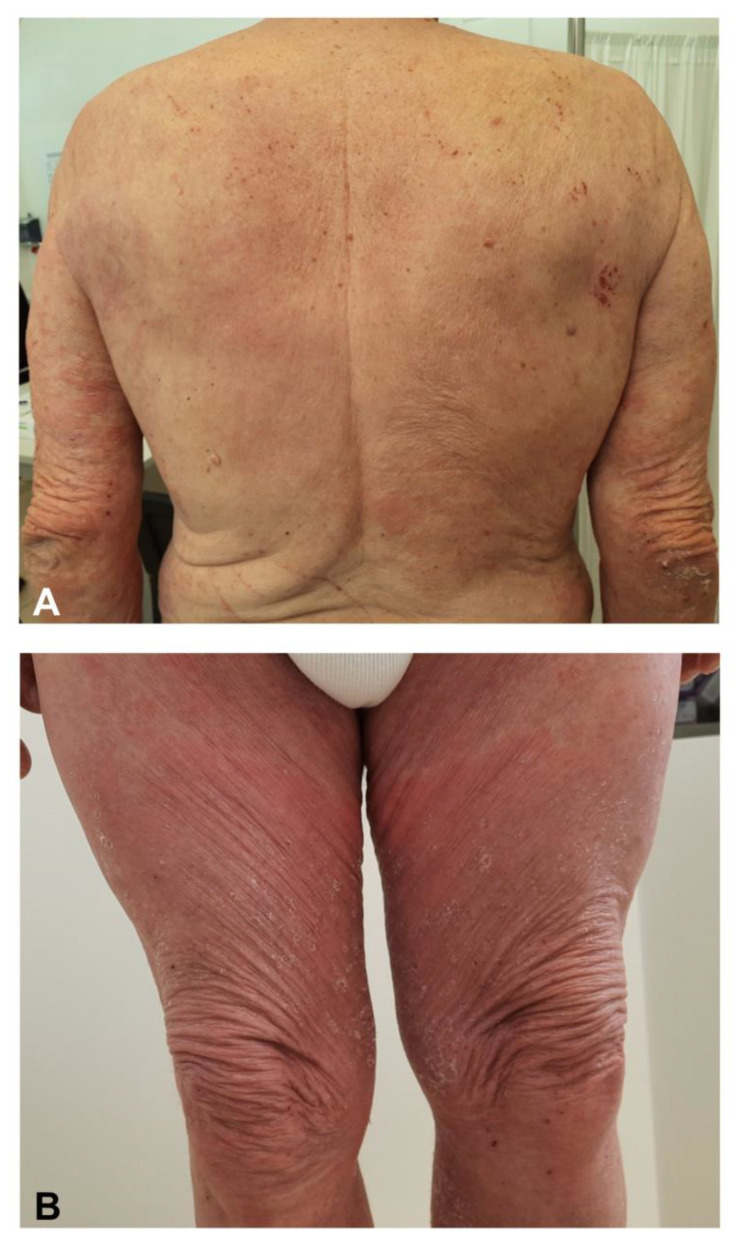
Diffuse bilateral eczematous lesions with overlying excoriation of the back, arms, and legs on a patient without a history of atopic dermatitis following the AZD1222 vaccine (71-year-old man) (**A**,**B**).

Diffuse or localized eczematous dermatitis in patients apparently without a history of atopic dermatitis have been reported following SARS-CoV-2 vaccination. The clinical findings are eczematous erythematous vesicular patches and papules localized on the back, arms, and legs [23,42,46] (Figure 5). Lesions tend to be bilateral but not as symmetrical as exanthematous reactions. The histopathology shows a typical spongiotic dermatitis and lympho-histiocytic infiltrate with eosinophils [20,46]. The administration of the second dose could be followed by the flare of eczematous lesions in those patients who experienced eczema after the first dose. Eczema can be controlled by systemic or topical glucocorticosteroids [20,46].

### 3.8. Autoimmune Bullous Reactions

Autoimmune bullous reactions following mRNA vaccines are uncommon, and few cases of autoimmune subepidermal bullous diseases have been reported. Their clinical presentations show itchy, urticated, erythematous plaques and tense bullae developing 3–21 days after the first or second dose. Most cases described fulfilled the diagnostic criteria for bullous pemphigoid or, rarely, a linear immunoglobulin A bullous dermatosis [20,47]. In a series of 12 new onset subepidermal blistering eruptions (including eight confirmed bullous pemphigoid) following the first or second dose of mRNA vaccination, the mean time to onset was 7 days [48]. Cases of severe pemphigus vulgaris following a few days after the BNT162b2 vaccination were also described [49].

### 3.9. Severe Cutaneous Adverse Reactions (SCARs)

Fortunately, SCARs following COVID-19 vaccinations are rare and include cases of AGEP, drug reactions with eosinophilia, and systemic symptoms (DRESS) and SJS/TEN. In these cases, no other drug-related triggers were identified. Annabi et al. reported a case of AGEP in a 43-year-old woman three days after an AstraZeneca/Oxford immunization [18]. The patient was treated with topical glucocorticosteroids with complete resolution and received a different type of vaccine (Pfizer/BioNTech) as the second dose. Lospinoso et al. described a 74-year-old man who developed a diffuse pustular rash accompanied by systemic symptoms and eosinophilia, compatible with DRESS, the day after the Janssen vaccine. The patient was treated with 20 mg/d of oral prednisone and topical glucocorticosteroids with complete remission [50]. Dash et al. reported a case of SJS in a 60-year-old man three days after the first dose of AstraZeneca/Oxford. The patient was successfully treated with 300 mg/day of cyclosporine, with complete resolution in one week. The second dose of the vaccination was deferred [51]. Bakir et al. reported another case of TEN in a 46-year-old woman one week after the first dose of the Pfizer/BioNTech vaccine. [52]. One case of extensive erythema-multiforme in a 62-year-old man following a second dose of the Moderna vaccine was described. The patient developed erythema and blisters on the trunk, limbs, and genitalia. The blistering rash appeared after the first dose and worsened after the second dose [53]. Finally, one case of extensive bullous fixed drug eruption was reported the day after the second dose of the Moderna vaccine [54].

### 3.10. Other Dermatoses

One case of Sweet syndrome accompanied by acute encephalitis was described in a 77-year-old man after the first dose of the mRNA-1273 vaccine [55]. There are sporadic reports of fixed drug eruptions [6,18,56]. Two middle-aged women developed a rosacea-like eruption 4 to 5 days after Ad26.CoV2.S and BNT162b2 vaccination [57]. In some patients, COVID vaccination may induce a new onset of psoriatic disease, presenting as chronic plaque psoriasis [58,59], palmoplantar psoriasis [60], guttate psoriasis [61], generalized pustular psoriasis [62], and even pityriasis rubra pilaris [63].

### 3.11. Flare of Existing Dermatoses

Exacerbations of distinct chronic cutaneous dermatoses have been reported after the mRNA and adenovirus-vectored vaccines (Table 2). Several flares of psoriasis have been clearly documented, including cases of new onset psoriatic arthritis [6,7,58,59]. The development of erythematous–desquamative plaques of psoriasis after the mRNA and Ad26.CoV2.S vaccinations was described in a case series of 14 patients. Of these, nine had a preexisting history of psoriatic disease. Almost all individuals had an exacerbation of their disease relatively soon after the second vaccine dose in a mean time of 10 days [58]. Nonetheless, Kadali et al. demonstrated that, in a group of 131 patients with psoriatic arthritis, the postvaccination measures of disease activity PASI were stable in most cases [24]. However, about 20% of 117 patients had a worsening of their DAPSA score [24]. Other conditions potentially worsened by the COVID-19 vaccination are atopic dermatitis [6,20,42], lichen ruber planus [6], chronic spontaneous urticaria [64], bullous pemphigoid [65], pemphigus vulgaris [65], pityriasis rubra pilaris [66], urticarial vasculitis, cutaneous small-vessel vasculitis [67], erythema multiforme [68], and Darier’s disease [69]. Finally, one case of transition from subacute cutaneous lupus erythematosus into systemic lupus erythematosus was also described [70]. Some cases of radiation recall that the phenomenon was reported 5 to 6 days after the mRNA and adenovirus-vectored vaccines. The patients showed typical acute inflammatory reactions localized to the previously irradiated skin that was successfully treated with local glucocorticosteroids [71,72]. Two cases of local Bacillus Calmette–Guérin (BCG) inflammation following mRNA vaccination were described 24 h after the second dose. Clinically, BCG inflammation presented as a painful, erythematous, and indurated plaque at the BCG scar site [73].

Several cases of herpes simplex virus (HSV) and varicella zoster virus (VZV) reactivation have been documented [6,7,74,75,76,77]. In a large study of 405 cutaneous reactions, VZV and HSV reactivations were 10% and 3% of the cases, respectively. Herpetic reactivations developed more commonly after the first dose, with a mean time ranging from a mean 5–7 ± 4–6 days (standard deviation) [6]. 

## 4. Discussion

Dermatologists can play an important role during the current mass vaccination campaigns addressing properly adverse cutaneous reactions secondary in the COVID-19 vaccines. The erroneous interpretation of skin reactions may expose some patients to the risk of severe reactions or may exclude some others from receiving a second dose unjustifiably. We distinguished two main categories of cutaneous reactions, flares of preexisting dermatoses and new onset reactions, and classified the latter into distinct morphological patterns. Local reactions are the commonest cutaneous reactions, particularly with mRNA-based vaccines, but they can also be elicited by adenovirus-vectored vaccines [78]. Apart from the local side effects, most cutaneous reactions secondary to SARS-CoV2 vaccination reflect those associated with COVID-19. To explain the filler reactions, it was hypothesized that the COVID-19 spike protein evokes a proinflammatory response in the location of dermal hyaluronic acid fillers through the blockade of a cutaneous ACE2 inhibitory pathway [17]. The mechanisms may involve a Th1 imbalance promoting a CD8+T-cell-mediated reaction to incipient granulomas around residual hyaluronic acid particles. Exanthemas (morbilliform or maculopapular, varicella-like, or papulovesicular); vascular lesions (purpura/petechiae, livedoid, and chilblain-like); and urticarial are morphological patterns that are already associated with SARS-CoV-2 infection [79]. Hence, common immunopathological mechanisms, including host immune activation against viral particles, rather than direct viral damage, may be involved [6,7,21]. Polyethylene glycol-2000 (PEG) and polysorbate 80 are the two main potential allergenic/immunogenic excipients in the COVID-19 vaccines. PEG-2000 is found in the Pfizer/BioNTech and Moderna vaccines and polysorbate 80 in the Oxford/AstraZeneca and Johnson & Johnson vaccines. They may play a role in eliciting urticaria through both immediate and delayed hypersensitivity reactions [80]. Other more uncommon reactions, such as erythromelalgia and pityriasis rosea-like rash, have also been reported following influenza and hepatitis B vaccinations. Finally, the vaccination may induce an immunomodulation that allows VZV to escape from its latent phase and elicit herpes zoster [75].

Interestingly, a higher proportion of cutaneous reactions was described in women, but it is still not clear whether this reflects a selection or publication bias or women’s greater reactogenicity to vaccines [4,6]. According to Català et al., a history of atopy or chronic urticaria does not predispose women to SARS-CoV-2 vaccine cutaneous reactions, even if flares of the diseases are possible [6].

We found that most of the cutaneous reactions were mild, skin-limited, and characterized by a rapid resolution. Cutaneous reactions to SARS-CoV-2 vaccines reflect the patterns observed with other vaccines [33]. For example, local and delayed skin reactions, such as urticaria, maculopapular, or nonspecific skin rashes, can be commonly found after the injection of vaccines containing toxoids and the hepatitis B virus vaccine [80]. Patients with mild or moderate cutaneous reactions should undergo the second dose and, in selected cases (i.e., mastocytosis), may benefit from a prophylactic premedication (i.e., antihistamines and oral or topical glucocorticoids) [81,82]. Administering a different vaccine type for the booster dose may be reasonable to reduce the risk of relapse of the previous moderate-to-severe reactions [18]. In general, there are no contraindications to vaccinate populations with allergic diseases such as atopic dermatitis or chronic urticaria [82,83]. In the case of an acute flare of eczema/chronic urticaria, patients may be vigorously treated, and vaccination should not be postponed [82]. Conversely, severe reactions like SCARs and severe immediate hypersensitivity reactions (i.e., anaphylaxis) are potentially life-threatening and represent contraindications for a second vaccine dose [83,84]. It is not clear whether using a different vaccine type for the booster injection may be advisable in such cases [18]. Precautions should be taken in the case of a history of anaphylaxis and in patients with systemic mastocytosis [81,84].

This review may be burdened by some limitations, including the nonsystematic method. Adverse events after SARS-CoV2 vaccination are being increasingly reported, reflecting a special interest during the pandemic. However, most of the studies are small case series burdened by publication biases that do not allow the drawing of general conclusions with high levels of evidence, such as estimating the prevalence of the different cutaneous reactions. In addition, many cases of flare-up of chronic dermatoses are not published, as the interest in the common and non-severe reactions is diminishing.

In conclusion, cutaneous reactions to the COVID-19 vaccination resemble common cutaneous drug eruptions and cutaneous manifestations of COVID-19. Dermatology’s perspective on the COVID-19 mass vaccination campaign is faceted and important in order to drive clinicians to properly address vaccination cutaneous reactions and to reassure patients.

## Figures and Tables

**Table 1:** Patterns of new onset adverse skin reactions associated with SARS-CoV-2 vaccinations.

Pattern	Subtype	Reported Associated Vaccines
Injection site reactions	Immediate local reaction	All
	Delayed local reaction	
Filler reactions	-	Pfizer/BioNTech, Moderna
Exanthemas	Erythematous confluent	All
	Maculopapular	
	Papulovesicular	
	Pityriasis rosea like	Pfizer/BioNTech, Moderna, AstraZeneca/Oxford
	SDRIFE	Pfizer/BioNTech
	Annular	Johnson & Johnson/Janssen
Vascular lesions	Leucocytoclasic vasculitis	All
	Urticarial vasculitis	Pfizer/BioNTech
	Purpura/petechiae	All
	Livedo racemosa	Pfizer/BioNTech
	Chilblain-like	Pfizer/BioNTech, Moderna
	Erythromelalgia	Pfizer/BioNTech, Moderna
Urticaria	Wheals and/or angioedema	Pfizer/BioNTech, Moderna, AstraZeneca/Oxford
Eczematous dermatitis	-	Pfizer/BioNTech, Moderna
Autoimmune bullous reactions	Subepidermal bullous diseases	Pfizer/BioNTech, Moderna
	Pemphigus	Pfizer/BioNTech
SCARs	AGEP	AstraZeneca/Oxford
	DRESS	Johnson & Johnson/Janssen
	SJS/TEN	Pfizer/BioNTech, AstraZeneca/Oxford
	EM major	Moderna
	Extensive FDE	Moderna
Other dermatoses	Sweet syndrome	Moderna
	Rosacea-like eruption	Pfizer/BioNTech, Johnson & Johnson/Janssen
	Psoriasis (plaque, guttate, palmoplantar, and generalized pustular)	Pfizer/BioNTech, Moderna, Johnson & Johnson/Janssen
	Pityriasis rubra pilaris-like eruption	Pfizer/BioNTech

SDRIFE: Symmetrical Drug-related Intertriginous and Flexural Exanthema; SCARs: Severe cutaneous adverse reactions; SJS: Stevens-Johnson syndrome; TEN: toxic epidermal necrolysis; AGEP: acute generalized exanthematous pustulosis; DRESS: drug reaction with eosinophilia and systemic symptoms; FDE: fixed drug eruption; EM: erythema multiforme.

**Table 2:** Preexisting dermatoses flared by the SARS-CoV-2 vaccinations.

Type	Subtype	Reported Associated Vaccines
Immuno-mediated dermatoses	Chronic plaque psoriasis	Pfizer/BioNTech, Moderna, Johnson & Johnson/Janssen
	Atopic dermatitis	Pfizer/BioNTech, Moderna
	Lichen ruber planus	Pfizer/BioNTech, Moderna
	Chronic spontaneous urticaria	Moderna
	Bullous pemphigoid	Pfizer/BioNTech, Moderna
	Pemphigus vulgaris	Pfizer/BioNTech, Moderna
	Pityriasis rubra pilaris	AstraZeneca/Oxford
	Cutaneous small-vessel vasculitis	Pfizer/BioNTech
	Erythema multiforme	Pfizer/BioNTech
	Darier’s disease	AstraZeneca/Oxford
	Systemic lupus erythematosus	AstraZeneca/Oxford
	Radiation recall phenomenon	Pfizer/BioNTech, AstraZeneca/Oxford
	BCG inflammation	Pfizer/BioNTech, Moderna
Infectious dermatoses	HSV reactivation	All
	VZV reactivation	

BCG Bacillus Calmette–Guérin, HSV Herpes simplex virus, and VZV Varicella zoster virus.

## Data Availability

Data sharing not applicable.
